# Conductor Losses in Radiofrequency Coils for Magnetic Resonance below 3T: Estimation Methods and Minimization Strategies

**DOI:** 10.3390/s23125586

**Published:** 2023-06-14

**Authors:** Giulio Giovannetti, Alessandra Flori, Francesca Frijia

**Affiliations:** 1 Institute of Clinical Physiology, National Research Council (CNR), Via G. Moruzzi 1, 56124 Pisa, Italy; 2 U.O.C. Bioingegneria e Ingegneria Clinica, Fondazione Toscana Gabriele Monasterio, Via G. Moruzzi 1, 56124 Pisa, Italy; alessandra.flori@ftgm.it (A.F.); f.frijia@ftgm.it (F.F.)

**Keywords:** Magnetic Resonance, RF coils, conductor losses, skin effect, signal-to-noise ratio

## Abstract

The design of optimized radiofrequency (RF) coils is a fundamental task for maximizing the signal-to-noise ratio (SNR) in Magnetic Resonance Imaging (MRI) and Magnetic Resonance Spectroscopy (MRS) applications. An efficient coil should be designed by minimizing the coil noise with respect to the sample noise, since coil conductor resistance affects data quality by reducing the SNR, especially for coils tuned to a low frequency. Such conductor losses strongly depend on the frequency (due to the skin effect) and on the conductor cross-sectional shape (strip or wire). This paper reviews the different methods for estimating conductor losses in RF coils for MRI/MRS applications, comprising analytical formulations, theoretical/experimental hybrid approaches and full-wave simulations. Moreover, the different strategies for minimizing such losses, including the use of Litz wire, cooled and superconducting coils, are described. Finally, recent emerging technologies in RF coil design are briefly reviewed.

## 1. Introduction

Magnetic Resonance Imaging (MRI) is one of the most important medical imaging techniques employed for different organ and tissue disease diagnoses and follow-up, thanks to its noninvasive and high-sensitivity skills to image the human body and animal models. In addition, Magnetic Resonance Spectroscopy (MRS) of ^1^H and other nuclei such as ^19^F, ^23^Na, ^31^P and ^13^C provide metabolic function and chemical process information. Both MRI and MRS employ a static magnetic field (B_0_) jointly with radiofrequency (RF) pulses and gradients for image acquisition. The RF field (B_1_) serves to excite the nuclei within biological tissues and is generated by a transmit coil, while the re-emitted signal is collected by a receive coil. RF coils are therefore key components in Magnetic Resonance (MR) systems, since image quality strongly depends on them. In particular, transmit coils have to cover a wide field of view (FOV) with high magnetic field homogeneity, while receive coils have to provide high signal-to-noise ratio (SNR) in acquired data [1]. RF coils can be categorized into volume and surface coils [2]. Volume coils enclose the object to be imaged and are employed for producing a uniform magnetic field in the region of interest (ROI), while surface coils, constituted by single or multiple loops, have high sensitivity near the patient surface and provide a higher SNR with respect to volume coils, although with a relatively poor field uniformity. RF coils in phased-array configurations permit a large region of sensitivity, similar to volume coils, jointly with a high SNR, usually associated withwith surface coils [3]. The individual coil images acquired by the single array elements are then combined into a single composite image with a full FOV [4]. In general, RF coils have to be adapted to a specific goal and to the sample sizes, and, in the meanwhile, they have to guarantee a good performance with slightly different sample geometries. Therefore, an accurate design process is necessary for RF coil performance optimization. Such a process must include the choice of the coil conductor geometry, ensuring the minimization of the conductor losses with respect to the sample noise, which is a constraint, especially for low-frequency MR applications [5]. More generally, for an optimal coil design, it is important to know how the conductor’s cross-geometry choice affects the RF coil’s overall performance.

**Figure 1:**
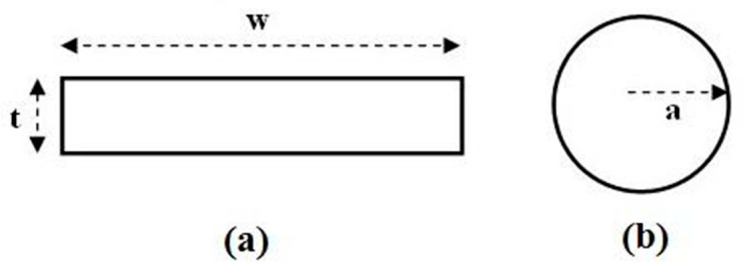
RF coil conductor typologies: strip (**a**) and wire (**b**).

Dependent on their cross-sectional shape, conductors used for RF coil construction can be categorized into strips (rectangular shapes, characterized by width *w* and thickness *t*) and wires (cylindrical rod shapes, defined by their radius *a*), as depicted in Figure 1.

This review is organized as follows: after a brief description of the RF coil performance in terms of losses (Section 2) and how these affect the SNR data (Section 3), a mention of the skin effect on coil conductors (Section 4) is introduced to the core review topic. In particular, we summarize the papers that investigated conductor geometry effects and compared strip and wire conductor performances in RF coils for MR applications, using both theoretical (Section 5) and theoretical–experimental “hybrid” (Section 6) approaches. Moreover, a focus on full-wave simulations (Section 7) and experimental measurements for conductor loss estimations (Section 8) are included. Finally, Section 9 describes the use of Litz wire, cooled and superconducting coils, while, in Section 10, the different and emerging strategies for minimizing conductor losses are described. Although the last two sections provide some details regarding magnetic field strength > 3T, we believe these can be employed even for the design of RF coils tuned at lower frequencies.

## 2. RF Coil as RLC Circuit

**Figure 2:**
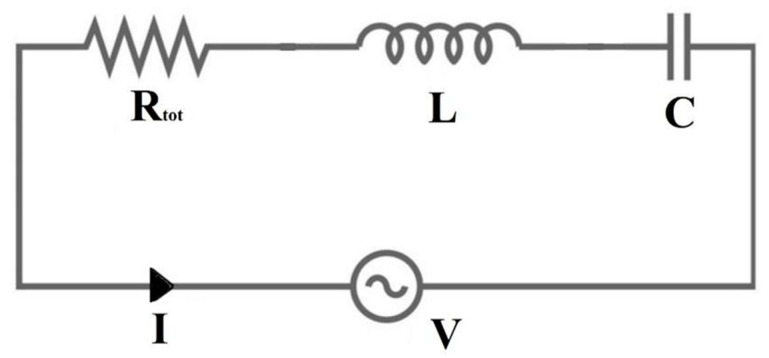
RLC equivalent circuit of an RF coil.

RF coil can be schematized with an equivalent RLC circuit, where the flowing current *I* is maximized at the *f*_0_ Larmor frequency [6] (Figure 2).

The reciprocity theorem [7] defines *V* as the voltage source (for the transmit coil) or the sample-induced voltage (for the receive coil). *L* is the coil inductance, which takes into account the energy stored in the magnetic field, while *C* is the capacitance mainly resulting from discrete capacitor contributions.

The term *R_tot_* represents the sum of all the resistances associated with the different loss mechanisms within the conductors and the sample [1]:

(1) $$R_{tot}=R_{coil}+R_{sample}+R_{extra}$$

*R_coil_* takes into account the coil conductor losses, and *R_sample_* is the sample losses caused by RF currents, induced by the fluctuating magnetic field and by the electric fields in the sample, mainly generated by the capacitors. Finally, *R_extra_* comprises the radiative, tuning capacitor and soldering losses. A quantitative measure of the circuit quality is provided by the coil quality factor, expressed in terms of the circuit parameters [8]:

(2) $$Q=\frac{2\pi f_{0}L}{R_{tot}}=\frac{1}{R_{tot}}\sqrt{\frac{L}{C}}$$

A common parameter for coil performance evaluation is the ratio *r* between the quality factor of an empty coil (*Q_unloaded_*) and the coil with the sample (*Q_loaded_*) [9]:

(3) $$r=\frac{Q_{unloaded}}{Q_{loaded}}=1+\frac{R_{sample}}{R_{coil}+R_{extra}}$$

The optimal coil design has to be performed by minimizing the coil noise with respect to the sample noise in order to provide the maximum SNR, since $SNR\alpha \sqrt{1-\frac{1}{r}}$ [10].

Finally, another parameter characterizing RF coil performance is the sensitivity, defined as the *B*_1_ magnetic field induced by the RF coil at a given point per unit of supplied power *P*, as follows [1]:

(4) $$\eta =\frac{B_{1}}{\sqrt{P}}$$

The reciprocity theorem [7] uses Equation (4) for characterizing both the transmit and the receive performances of an RF coil.

## 3. The SNR

The *SNR* is an accepted standard parameter for quality evaluation in MR experiments and depends on the hardware, particularly the main field strength and RF coils, on the acquisition sequence parameters and on the tissue relaxation properties. Analytically, the *SNR* at the observation point *P* (*SNR_P_*) can be defined as the ratio between the induced RF signal and the root mean square (RMS) of the thermal noise voltage measured at the coil terminals [11]:

(5) $$SNR_{P}=\frac{2\pi f_{0}MVB_{P}}{\sqrt{4kT\Delta fR_{tot}}}$$

where *M* is the magnetization, *V* is the voxel volume, *B_P_* is the received coil magnetic field per unit current at the observation point *P*, *k* is the Boltzmann constant, *T* is the absolute resistance temperature and *Δf* is the receiver bandwidth.

An estimation of the *SNR* dependence on the frequency can be calculated by considering a sample with *d* as its linear size. In particular, by taking into account the RF current distribution in the coil conductor cross-section, $R_{coil}\approx f^{\frac{1}{2}}d^{-1}$, whereas, in the near-field assumption, $R_{sample}\approx f^{2}d^{3}$ [12]. At low RF frequencies, the *SNR* is mainly determined by the coil losses, and Equation (5) indicates that $SNR\approx f_{0}^{\frac{7}{4}}d^{2}$ [13]. In this frequency range, the *SNR* can be improved with the use of high *Q* factor RF coils, since for a coil tuned at *f_0_* frequency, the $SNR\approx \sqrt{Q}$ [14], which can be built by using high-quality capacitors and optimized conductor cross-geometry. At high RF frequencies, the sample losses are dominant, and $SNR\approx f_{0}d^{\frac{1}{2}}$. This last condition generally occurs in clinical fields (>0.5 T) [5].

## 4. Current Distribution in Coil Conductors

The solution of the Maxwell’s equations in complex form leads to the assumption that, unlike a direct current (DC), an alternating current (AC) flowing in a conductor is not uniformly distributed along its cross-section but is confined to a region near the surface which thickness (penetration depth) *δ* can be calculated as [15]:

(6) $$\delta =\sqrt{\frac{\rho }{\pi f\mu \;}}$$

where *ρ* is the conductor resistivity (*ρ* = 1.68 × 10^−8^ m·Ω for copper), *f* is the coil tuning frequency and *μ* is the conductor permeability (4π × 10^−7^ Henry per meter). From a mathematical point of view, *δ* is the distance at which the current density vector amplitude decreases to 1/*e* of its value at the boundary surface, but we can assume that the current flows in a peripheral layer of thickness *δ*, with the current density uniform in this area and zero at the conductor center. In practice, the conductor volume crossed by the RF current is limited by the penetration depth value, and this phenomenon is called the “classical skin effect” [16].

The coil conductor resistance can be calculated by taking into account the conducting pathway geometry. In particular, it can be estimated with the classic formula [17]:

(7) $$R_{cond}=\frac{\rho l}{S}$$

where *l* is the total conductor length, and *S* the cross-sectional area in which the current actually flows, according to the penetration depth value of Equation (6).

## 5. Theoretical Approaches for Conductor Resistance Calculation

In a wire conductor (Figure 1b), when its radius *a* is much greater than *δ*, the conductor losses for the unit length can be estimated as follows:

(8) $$R_{wire‐clas}=\frac{\rho }{2\pi a\delta }$$

which contains a dependence on the frequency through the penetration depth, as in Equation (6).

In a strip conductor width *w* (Figure 1a), if its thickness *t* is greater than two times the penetration depth size, the conductor resistance for the unit length can be calculated as:

(9) $$R_{strip‐clas}=\frac{\rho }{2w\delta }$$

Otherwise, the current flows in the total conductor cross-sectional area, and the conductor resistance has to be evaluated as:

(10) $$R_{strip‐clas}=\frac{\rho }{tw\delta }$$

However, as reported by Frass-Kriegl [18], this is just a simplification for the strip conductor resistance evaluation, because it only considers the “classical” skin effect and neglects the fact that, in real cases, the current density mainly concentrates at the conductor points with the greatest curvatures. For example, in an elliptic cross-section conductor, the current density mainly gathers at the major axis ends with respect to the minor axis ends, and since the strip conductor can be schematized as the limiting case of a very thin elliptic cylinder, its current distribution is expected to be higher at the strip conductor edges. This is the “lateral skin effect” [19] and determines that the strip current distribution is less uniform than in the wire.

**Figure 3:**
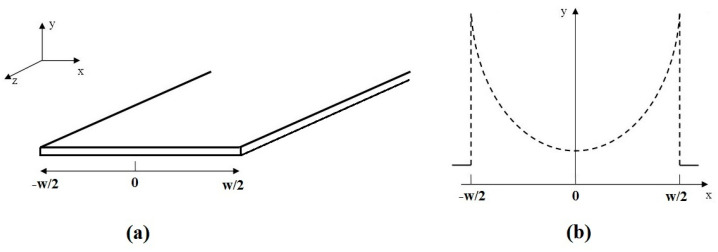
Infinitely long flat strip, (**a**) and the current distribution over its surface (**b**).

Carlson et al. [20] calculated the current distribution in an infinitely long flat strip conductor by solving Maxwell’s equations with boundary conditions by taking into account a perfectly conducting conductor approximation and imposing the condition that the fields vanish at infinity. With the assumption that the surface current flows in the *z* direction and that the current density function *J* is independent along the *z* (long wavelength assumption) and along the *y* (thin strip approximation) axes, the surface current for a strip of width *w* much thicker than the skin depth can be calculated as (Figure 3a)

(11) $$J\left(x\right)=\frac{I}{2\pi }\frac{1}{\sqrt{{\left(\frac{w}{2}\right)}^{2}-x^{2}}}$$

where *I* is the total current magnitude, and −*w*/2 < *x* < *w*/2. By plotting Equation (11), it is possible to note the increase of the current distribution near the flat strip edge and the tendency to generate a uniform pattern near the conductor center (Figure 3b).

Schmidt et al. [21] proposed an equation to find the *d_w_* wire conductor equivalent diameter that provides the same resistance of a strip conductor (with width *w* and thickness *t*):

(12) $$d_{w}=\frac{w}{1+1.13\log_{10}\left(\frac{w}{t}\right)}$$

This equation, valid only when *w* is much smaller than the wavelength and *t* is much thicker than the skin depth, indicated that the strip resistance is equal to that of a wire conductor with a diameter of about 1/4 of the strip width.

Terman et al. [22] proposed two expressions useful to calculate the high-frequency resistance per unit length (in Ω/cm) for different conductor typologies at frequency *f* (expressed in Hz). The first equation estimated such resistance for a wire with a diameter *d* (in cm) as

(13) $$r_{w}=10^{-9}\frac{83.2\sqrt{f}}{d}$$

While, for a strip with width *w* and thickness *t* (in cm), the following expression can be used:

(14) $$r_{s}=10^{-9}K\frac{261\sqrt{f}}{2\left(w+t\right)}$$

where *K* is a constant that depends on the *w/t* ratio, according to a plot shown in [22] for the range 1 < *w/t* < 100. The results provided by Equation (14) are accurate only when *t* is much greater than twice the skin depth value.

Mispelter et al. [23] discussed an approximated formulation for a strip resistance evaluation for the unit length, starting from Terman’s diagram [22] and valid when *w* > 10*t*:

(15) $$r_{s}=\frac{261\sqrt{f}}{2\left(w+t\right)}\left(1+0.54log_{10}\left(\frac{w}{t}\right)\right)$$

where the frequency *f* is expressed in MHz, *w* and *t* are expressed in mm and *r_s_* is expressed in mΩ/m.

Gerling et al. [24] approached the skin effect in strip conductors used for the development of hybrid and pure electric drives in the automotive industry sector, in which high frequencies are employed for reaching a high maximum speed. Starting from Maxwell’s equations, the analytical calculations were performed by applying a specific edge condition, taking into account the special behavior of the electromagnetic field at the strip edges, based on the fact that the electromagnetic energy in any finite domain must be finite. The exact solution for current density distribution dependent on the temperature and material was presented only for an arbitrary strip geometry and a low frequency (under 1 kHz) or for a symmetric conductor geometry (width = thickness) and arbitrary frequency. For the arbitrary geometry and frequency (but below 20 kHz), the discussed solution was only an approximation, but the results showed that, when the frequency increased, the current was displaced more and more to the strip corners.

Guo et al. [25] proposed the current distribution evaluation inside a strip by means of a numerical solution provided by a surface integral equation (SIE) formulation, which permitted two separate equivalent situations. In the exterior region, the strip was modeled as an electric surface current radiating in the free space, while, in the interior region, the strip was modeled as an electric surface current that radiates in a homogeneous space with the conductivity of the strip conductor. After the enforcement of the electric (*E*) and magnetic (*H*) field continuity at the strip surface, the AC resistance was calculated from the power dissipated inside the strip, estimated by the Poynting vector flux over the strip surface as:

(16) $$R_{AC}=\frac{Re\oint_{S}^{}\left(E\times H\right)ds}{{\left|I\right|}^{2}}$$

where *I* is the total current, evaluated with the line integral of the magnetic field around the strip perimeter. The results indicated that the current distribution over the conductor cross-sectional area caused the *R_AC_*/*R_DC_* ratio to increase due to the skin effect, because portions of the conductor were not fully effective in carrying the current. Moreover, the same paper showed a diagram computing the ratio *R_AC_*/*R_DC_* for strip (width *w* and thickness *t*) and wire (radius *r*) conductors with the same cross-sectional area (*wt* = *πr*^2^) as a function of the square root of the frequency for different ratios *w*/*t* between 1 and 32. Such results were in good agreement with the experimental one described in [22] and were used for a digital circuits skin effect analysis operating at rates of hundreds of MHz [26].

**Figure 4:**
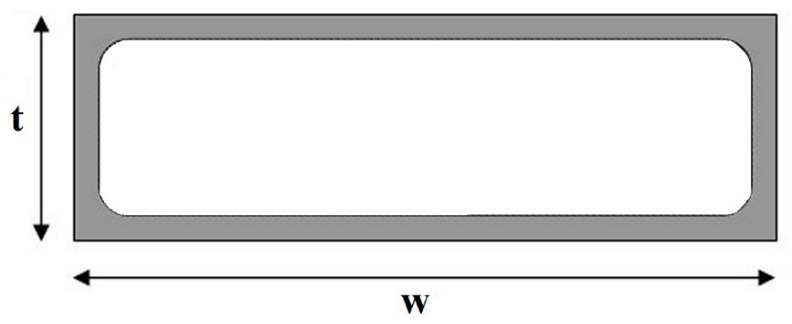
High-frequency current path (grey area) in a strip conductor.

Faraji et al. [27] proposed the AC resistance estimation of a microstripline used in a monolithic microwave integrated circuit (MMIC) in which the insertion loss was strongly affected by such resistance. The formulation was performed by examining the strip conductors with different aspect ratios *w*/*t* = 1, 2, 5 and 6 at various normalized frequencies. The analysis of the electric field distribution over the strip conductor cross-section showed that, at low frequencies, where the skin depth was of the order of the strip thickness (*t* < 2*δ*), an almost-uniform current distribution was observed, while, at high frequencies, when the skin depth was much smaller than the strip thickness (*t* > 4*δ*), the electric field distribution inside the conductor showed a nearly exponential decay. An approximate AC resistance calculation in the entire frequency range was then formalized by analyzing the strip cross-sectional area between the 0 and ∞ frequency range (see Figure 4):

(17) $$\underset{f\to 0}{\lim}R_{AC}=R_{DC}=\frac{\rho }{wt}$$

(18) $$\underset{f\to \infty }{\lim}R_{AC}=kR_{HF}=k\frac{\rho }{2\delta \left(w+t\right)}$$

where *R_HF_* is the AC resistance of a hollow tube with an equal circumference carrying a uniform current across its depth *δ*, while *k* is a correction factor that takes into account the existing edge field behavior.

As a result, the AC resistance can be estimated as:

(19) $$R_{AC}=\sqrt{\left[{\left(R_{DC}\right)}^{2}+{\left(kR_{HF}\right)}^{2}\right]}$$

Waldow et al. [28] approached the losses calculation in striplines of a rectangular cross-section employed in monolithic microwave integrated circuits when the metallization thickness became the same order as the skin depth.

The development of a numerical calculation method, based on a variational formulation of the skin effect problem, verified the experimental measurements published previously [29]; however, such a theory was validated in the frequency range of 1–20 GHz, much higher than those used in MRI.

## 6. A Theoretical–Experimental Hybrid Method

Giovannetti et al. [30] suggested a theoretical–experimental hybrid method, useful for distinguishing and quantifying the classical and lateral skin effect contributions to the conductor resistance in RF coils for MR applications. Two 7.5 cm radius circular loops were built by using a strip (0.45 cm width *w* and 40 µm thickness *t*) and a wire conductor (0.1 cm radius *a*), respectively, whose conductor cross-sectional size choices guaranteed the same coil *L* inductance value as according to [8].

(20) w=4.482·a

This equation, useful for finding the “equivalent strip width *w*” of a wire with a radius *a* in terms of the conductor inductance, can be easily derived by starting from the formula for the inductance calculation of a strip’s width *w* and length *l* [8]:

(21) $$L_{strip}=\frac{\mu l}{2\pi }\left(ln\frac{2l}{w}+\frac{1}{2}\right)$$

and from the formula for calculating the inductance of a wire’s radius *a* and length *l* [8]:

(22) $$L_{wire}=\frac{\mu l}{2\pi }\left(ln\frac{2l}{a}-1\right)$$

By equaling Equations (21) and (22) and assuming the same length *l* for the two conductors, some simple mathematical operations demonstrate Equation (20).

The strip coil resistance *R_strip-coil_* calculation was performed by summing the classical (*R_strip-clas_*) and lateral (*R_strip-lat_*) skin effect contributions:

(23) $$R_{strip‐coil}=R_{strip‐clas}+R_{strip‐lat}$$

where the first term was evaluated with Equation (9) or Equation (10), according to the strip thickness/penetration depth values relationship and by multiplying for the conductor total length (2π times the loop radius), as in Equation (7).

Conversely, due to the absence of conductor edges, the wire coil resistance *R_wire-coil_* is equal to the classical skin effect resistance provided by Equation (8) (and multiplied by the conductor length); therefore:

(24) $$R_{wire‐coil}=R_{wire‐clas}$$

Workbench measurements of the coil quality factors *Q* and the use of Equation (2) for the unloaded coils (*R_tot_* = *R_coil_* + *R_extra_*) estimated the total loss resistances for both coils as

(25) $$R_{strip‐tot}=\frac{2\pi f_{0}L}{Q_{strip}}$$

(26) $$R_{wire‐tot}=\frac{2\pi f_{0}L}{Q_{wire}}$$

By supposing that *R_extra_* was identical for both coils, due to their equivalence in terms of sizes and being tuned by using the same capacitors, this was estimated as

(27) $$R_{extra}=R_{wire‐tot}-R_{wire‐clas}$$

Successively, the strip coil resistance was calculated as

(28) $$R_{strip‐coil}=R_{strip‐tot}-R_{extra}$$

and, with Equation (23), the lateral skin effect contribution was estimated as

(29) $$R_{strip‐lat}=R_{strip‐coil}-R_{strip‐clas}$$

The workbench test results of the two coils, both tuned at 5.7 MHz, showed a better performance from the wire coil, providing a quality factor of 233 against a value of 146 for the strip coil, with a gain of 59%. Such behavior was imputable to a better current distribution inside the wire with respect to the strip, as predicted theoretically. However, the paper underlined the difficulty in handling wire conductors for coil building, which could require qualified mechanical personnel. Regarding the strip coil, the measurements indicated similar values for the two different skin effect contributions, the classical skin effect and the lateral skin effect resistance being equal to 51 and 49% of the strip coil resistance, respectively.

Successively [31], the two same circular coils were employed for evaluating the coil resistance at different tuning frequencies widely used in clinical scanners (21–128 MHz, corresponding to 0.5–3 T static fields).

**Figure 5:**
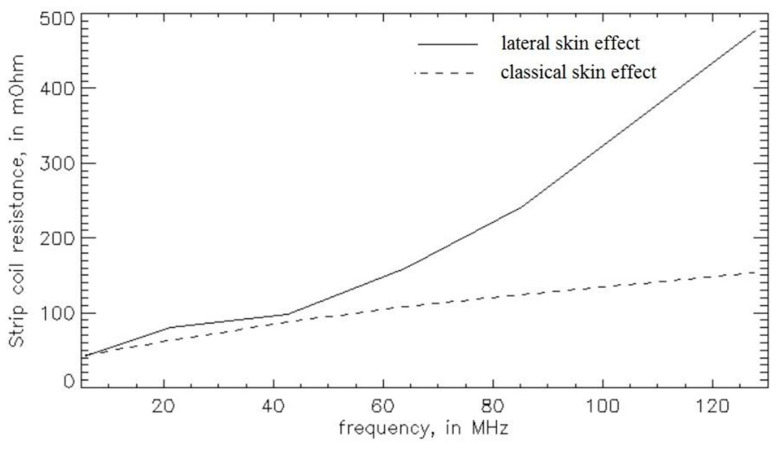
Strip coil skin effect resistance contributions dependent on the frequency.

Figure 5 depicts the plots of the different contributions to the skin effect resistance as a function of the strip coil tuning frequency; in particular, the classical and lateral skin effect resistances were calculated with Equations (9) and (28), respectively.

While the classical skin effect resistance plot described a similarity to the square root of the frequency, the lateral skin effect contribution showed a similarity very similar to the square of the frequency, showing that, in the frequency range routinely employed in a MR clinical scanner (21–128 MHz), this effect is the dominant mechanism that cannot be neglected, especially at high MR frequencies. The same paper compared the total resistance for strip and wire coils as a function of the tuning frequency, and the results confirmed the better performance of the coil constituted by a wire conductor; in particular, at 63.9 and at 127.8 MHz, the strip coil resistances, calculated as the sum of the classical and lateral skin effect resistance contributions, provided values of 267 and 630 mΩ, respectively, while the wire coil resistances results were 155 and 218 mΩ at 63.9 and 127.8 MHz, respectively.

## 7. Full-Wave Simulations

A simulation of AC conductors employed for electrical power transmission and distribution systems and for electronic devices was performed with a two-dimensional Finite Element Method (FEM) solver [32]. Experimental data acquired on wire conductors confirmed the accuracy of the FEM simulations, performed with a free license FEMM package, and its ability to provide more general results with respect to those provided by the analytical formulas, since FEM models can be applied to a wide range of cross-sections and frequencies. However, the approach was validated only in the range of 0–100 kHz, very far from the frequency routinely employed in MR. Conversely, Giovannetti et al. [33] proposed the application of FEM for performing wire coil loss estimations from 5.7 to 128 MHz. Simulations were performed on a 7.5 cm radius circular coil constituted by a 0.1 cm radius copper wire and by using CST MW Suite (CST-Computer Simulation Technology AG, Darmstadt, Germany). Coil impedances at the various frequencies were estimated by feeding the coil with an S-port and successively measuring the real parts of such impedances. Finally, since the solver permitted the power loss in the metal calculations, a separation of the coil conductor and radiation losses was performed. The FEM simulation results related to the coil conductor losses were initially compared with analytical calculations performed using Equation (8). At the lower frequencies (<63.9 MHz), the FEM and analytical calculations provided similar results, while, at higher frequencies, the results of the two different approaches diverged. In particular, the comparison showed that FEM predicted coil conductor losses at 5.7 MHz with a relative difference of <3% with respect to the analytical calculations, which became 13% at 63.9 MHz, and the relative difference increased to 58% at 127.8 MHz. This difference increase with the frequency was explained with the fact that the minimum tetrahedrons size employed in the simulations was not able to accurately take into account the skin effect at the higher frequencies.

**Figure 6:**
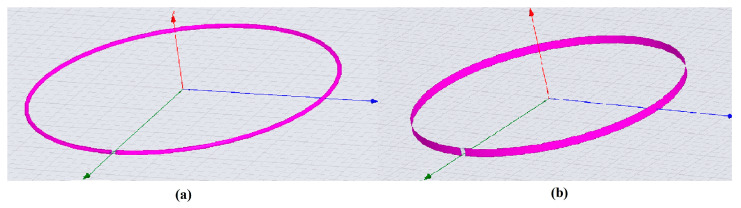
The 7.5 cm radius circular loops: (**a**) wire coil and (**b**) strip coil.

In a successive paper, the same authors [34] simulated two 7.5 cm radius circular coils: the first one was constituted by a 0.1 cm radius copper circular wire, identical to the one employed in the previous study [33], and a second coil constituted by a 0.45 cm width copper strip, which size guaranteed the same inductance of the circular wire coil, according to Equation (20). Such simulations were performed in the same frequency range of the previous work (5.7–127.8 MHz) and by using HFSS-FEM (Ansys, Canonsburg, PA, USA) (Figure 6).

While the circular wire coil HFSS-FEM simulations were in good agreement with the results obtained with CST-FEM in [33] (for example, only a 1.1% deviation in the conductor coil loss estimations at 63.9 MHz), the strip coil HFSS simulations underlined the higher losses of such coils, with an increase of 46% at 63.9 MHz and 56% at 127.8 MHz with respect to the wire coil.

The finite-difference time-domain (FDTD) method is mainly used for loaded coil magnetic field pattern estimations [35], Specific Absorption Rate (SAR) calculations [36] and sample-induced resistance estimations [37]. However, it is also employed for calculating losses in MR coil conductors. The preliminary results performed at 128 MHz showed that the conductor geometry, the computational mesh and the simulation software tool strongly affected the results, and underestimations of up to a factor of three resulted in the calculation of strip conductor losses.

A very recent paper [38] proposed the application of the FDTD method for separately calculating conductor and radiative losses in a circular loop constituted by a wire conductor from 21 to 128 MHz and with different conductor segmentation (non-segmented loop and loop segmented with eight ports). The simulations, performed with commercially available software XFdtd (Remcom, State College, PA, USA), employed an automatic nonuniform mesh (finer in the coil area) for minimizing the computational load and the simulation time. Other setups, which optimized the simulation accuracy, were a correction to the material conductivity for taking into account to the penetration depth and loss in copper for all frequencies, useful when the cell sizes were greater than the skin depth, and the use of perfect electric conductor (PEC) sheets placed to model the losses on the surface of each cross-sectional area of the coil gaps.

Coil conductor resistances at different frequencies of the non-segmented loop provided results similar to that obtained with the FEM simulation of a one-feed coil [33], with a relative difference below 4.35%. In this condition, if the non-segmented loop circumference approached a significant fraction of the wavelength, the coil started to act as a bent dipole, and a nonuniform current flowed along it. Conversely, the N = 8 segmented loop simulation results showed very good agreement with those obtained with the analytical calculations performed with Equation (8) (relative difference < 1.59%), since both simulations contemplated a uniform current along the coil path.

## 8. Experimental Measurements

Regarding the measurements performed for comparing strip and wire coil losses in [31], two 7.5 cm radius circular loops with the same inductance values, one constituted by a 0.1 cm radius wire and the other by a 0.45 cm width strip (see Equation (20)), were tested with a network analyzer. The coil quality factors (as defined in Equation (2)) were initially measured, and the results provided Q_s_ = 315 and Q_w_ = 345 for, respectively, the strip and wire coils, showing a 9.5% increase of the wire coil quality factor with respect to the strip coil value. Successively, the perturbing sphere method [39] was employed for the coil sensitivity measurements, according to Equation (4). Such measurements, performed at the loop centers and for both strip and wire coils tuned at 42.6 MHz, showed that the strip coil sensitivity result was 9.48 µT/W^1/2^, while the wire coil was 11.01 µT/W^1/2^, with a 28% increase with respect to the strip coil value. In a successive paper [34], the total resistance measurements performed on the same two coils, which included conductor losses, radiative losses and further resistive losses accountable to the solder joints between the coil and the cable for the connection with the analyzer, confirmed the higher losses of the strip coil. In particular, the strip coil total resistance was 300 mΩ at 63.9 MHz and 1020 mΩ at 127.8 MHz, while the wire coil total resistance was 260 mΩ at 63.9 MHz and 950 mΩ at 127.8 MHz. Such values underlined that the strip coil total resistance was greater than 15% at 63.9 MHz and 7% at 127.8 MHz with respect to the wire coil.

Regarding volume coils, Giovannetti [40] compared three lowpass birdcage coils with identical sizes (11 cm length, 13.4 cm diameter and 8 legs) and tuned at the same resonant frequency (7.66 MHz, which corresponded to a penetration depth of 23 µm) designed for being employed in a 0.18 T MR scanner.

**Figure 7:**
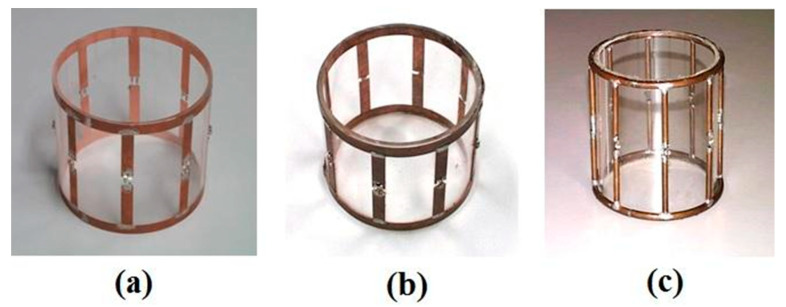
The three birdcages: (**a**) 35 µm thickness strip coil, (**b**) 800 µm thickness strip coil and (**c**) wire coil. Reprinted with permission from Ref. [40]. 2004, Wiley.

The first coil was built using a 1 cm width strip conductor with 35 µm thickness, the second coil with a 1 cm width strip conductor and 800 µm thickness, which is much greater than the penetration depth at the tuning frequency, as suggested by the literature [41], while the third coil employed a 2.25 mm radius wire conductor for maintaining the same conductor inductance value as according to Equation (20). The three built coils are shown in Figure 7.

The workbench test results of the three birdcages in terms of the *Q* factor, the *r* ratio between the unloaded and loaded (with a saline solution phantom) *Q* and the coil sensitivity are reported in Table 1.

The comparison between the two strip birdcages underlined that the use of a strip with a thickness much higher than the penetration depth permitted an overall coil performance increase (64% in *Q*, 14% in *r* and 23% in *η*) thanks to the conductor resistance reduction. However, as theoretically predicted, the wire birdcage provided the best performance, with a 28% increase in *Q*, 26% in *r*, and 22% in *η* with respect to the best strip birdcage.

## 9. Strategies for Minimizing the Conductor Losses

### 9.1. Litz Wire

**Figure 8:**
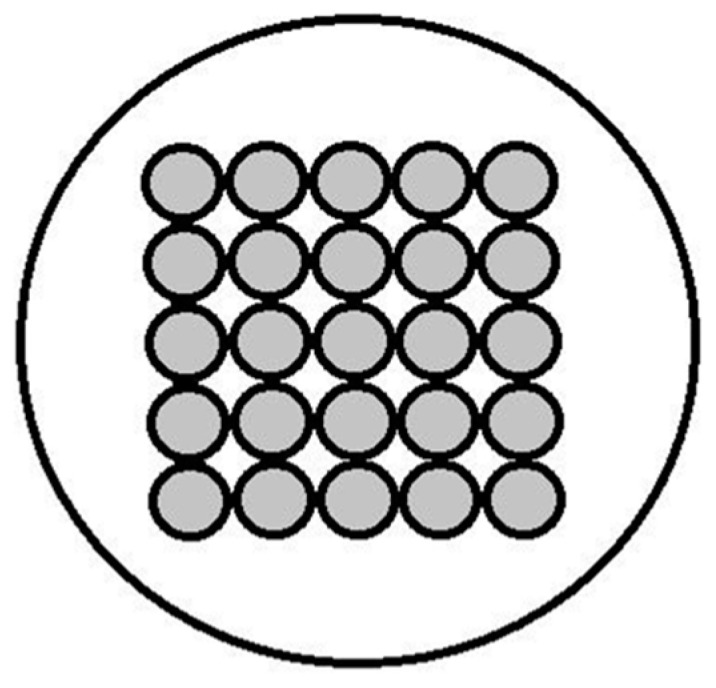
Sketch of the Litz wire cross-sectional area.

Litz wires consist of conductors made up of multiple individually insulated strands twisted or woven together. The strands are arranged in order to occupy all the positions within the conductor (Figure 8), thus providing equal distribution of the flowing current among the separated strands and a consequent reduction of the coil losses [42,43].

In Type 1 Litz wire, a set of strands twisted together forms one single conductor, whereas, in hybrid designs, multiple Type 1 conductors are woven together. The type of the Litz wire is usually selected depending on the specific application [44].

In general, we can distinguish three types of losses for Litz wires [45,46]:

1. Skin losses (deriving from skin effect) within each considered strand;
2. Proximity losses (due to the proximity effect) in the surrounding strands and nearby conductors;
3. Eddy losses (frequency-independent losses or DC losses) due to the environment of the coil (mainly determined by sample losses).

Litz wire optimization for power applications usually focuses on minimizing both DC and AC losses, while the design of MRI coils using Litz wires mainly deals with minimizing only RF (skin and proximity) losses [42].

In the previous sections, we have already analyzed skin losses resulting from the so-called “skin effect”. Proximity losses, on the other hand, result from the magnetic fields from nearby conductors (“proximity effect”), which reduce the effective cross-sectional area for a flowing current. Proximity losses decrease with increasing material resistivity and, in the case of Litz wires, can be reduced by providing sufficient spacing between conductors [44,45].

In more complex Litz wire windings, skin and proximity effects may be further divided into bundle-level and strand-level effects. Bundle-level effects relate to the current circulating along paths involving multiple strands and are determined by the overall diameter and the twisting pattern of the Litz wire. Strand-level effects, on the other hand, occur within individual strands. In this case, due to the large effective number of layers in a Litz winding, strand-level proximity effects prevail over skin effects [46].

When dealing with the design of RF coils for MRI applications, AC losses usually result in a reduced SNR and RF coil performance. As demonstrated in the literature, Litz wires represent a simple strategy to reduce skin and proximity effect losses, thereby leading to an increase in the SNR produced by RF coils at room temperature and to a potential increase of the available spatial resolution and sensitivity for imaging purposes. In fact, the use of multiple isolated strands of copper wire increases the effective cross-sectional area for the circulating current, thus reducing the skin effect. In parallel, the spatial distribution of the woven strands within the wire helps to reduce the proximity effects between strands.

For instance, Dominguez et al. [44] reported a reduction of the RF coil resistance by a factor of approximately two compared with conventional copper wire, depending on the construction of the Litz wire. According to Lofti et al. [47], a SNR gain of 1.5-fold due to coil resistance reduction by a practical Litz wire factor of 0.44 was reported, while Grafendorfer et al. [42] described a 2.2 SNR increase associated with the use of Litz wires in the construction of a saddle coil dedicated to human wrist MR images with a 0.4 T/5.7 MHz pre-polarized MRI scanner, as compared to a conventional coil design.

AC and DC losses and, in particular, the contribution provided by the skin or the proximity effect in a specific frequency range, can be calculated and visualized using simulation approaches and with numerical simulations [48,49]. In this context, the work by Roßkopf and colleagues [48] underlined the importance of including the behavior of the connector (identified as the source of inhomogeneous current distribution) in Litz wire simulations to greatly reduce losses during the construction.

Few geometrical factors determine the characteristics and the losses associated with the use of Litz wires for MRI coil construction. These factors should be considered when analyzing the behavior of Litz wires or when dealing with the optimization of the RF coil performance.

The ratio between the resistance to alternating currents (RAC) and the resistance to direct currents (RDC) in Litz wires can be estimated as [22]

(30) $$\frac{R_{AC}}{R_{DC}}=H+k{\left(\frac{nd_{s}}{d_{0}}\right)}^{2}G$$

where *H* is the ratio *R_AC_*/*R_DC_* of the individual strand when isolated (describing skin effect losses), *G* is a constant dependent on the neighboring wires proximity, *n* is the strand number in the cable, *d_s_* is an individual strand diameter, *d*_0_ is the cable diameter and *k* is a constant dependent on *n*.

The optimization of MRI coils using Litz wires mainly goes through the minimization of AC resistance (*R_AC_*) to reduce the total coil noise. According to theory, for a fixed cross-sectional area (*d*_0_), the total *R_AC_* is determined by the strand diameter *ds* and the number of strands *n*. Specifically, *ds* should be first set to a value smaller than the skin depth; afterwards, *n* should be optimized on the basis of the specific operational frequency of the coil to obtain a high performance (i.e., the optimized quality factor *Q* of the coil) [43,46,50].

Enpuku et al. [51] studied, both analytically and experimentally, the eddy current losses of a copper pickup coil made of Litz wire and cooled at 77 K for reducing thermal noise, suitable for ultra-low field NMR measurements. In the paper, the authors found that the coil eddy current losses strongly depend on the frequency (*f*), number of coil turns (*N*) and filament diameter of the Litz wire (*df*), leading to a large resistance at high working frequencies (*f* > 10 kHz). In particular, the coil resistance at high frequencies depends strongly on the filament diameter and is higher for larger *df* values. The coil resistance increase induced by the eddy current loss causes a degradation of the *Q* value and of the magnetic field noise of the coil that could be avoided by the proper selection of *N* and *df* coil parameters, especially using a Litz wire with a smaller *df*.

In another paper, Dominguez et al. [44] investigated the use of three different types of Litz wires for the construction of RF saddle coils suitable for MRI studies in phantom and rat lungs with hyperpolarized 3He and 129Xe gases at 73.5 mT (Larmor frequency = 2.385 MHz and 0.866 MHz, respectively). As reported in the paper, the different Litz wire characteristics (equivalent gauge (AWG), number of strands and operative frequency), as well as the selected number of coil turns, significantly affected the *Q* factor and the SNR provided by the RF coil. For this specific application, the best coil configuration was achieved using an AWG 18 Litz wire; in this case, 42% and 131% in vivo SNR improvements were obtained for 3He and 129Xe, respectively, as compared with conventional copper wire.

In parallel, Croon and colleagues [45] explored the use of single, as well as of six parallel, Litz wires for the construction of low-field solenoid coils (tuned at 356 kHz) and showed that the maximum coil quality could be obtained by equalizing the skin and proximity losses. In this case, if the strand diameter of the Litz wire is larger than the skin depth, the quality of the Litz wire coils is independent of the resistance of the conductor material.

**Figure 9:**
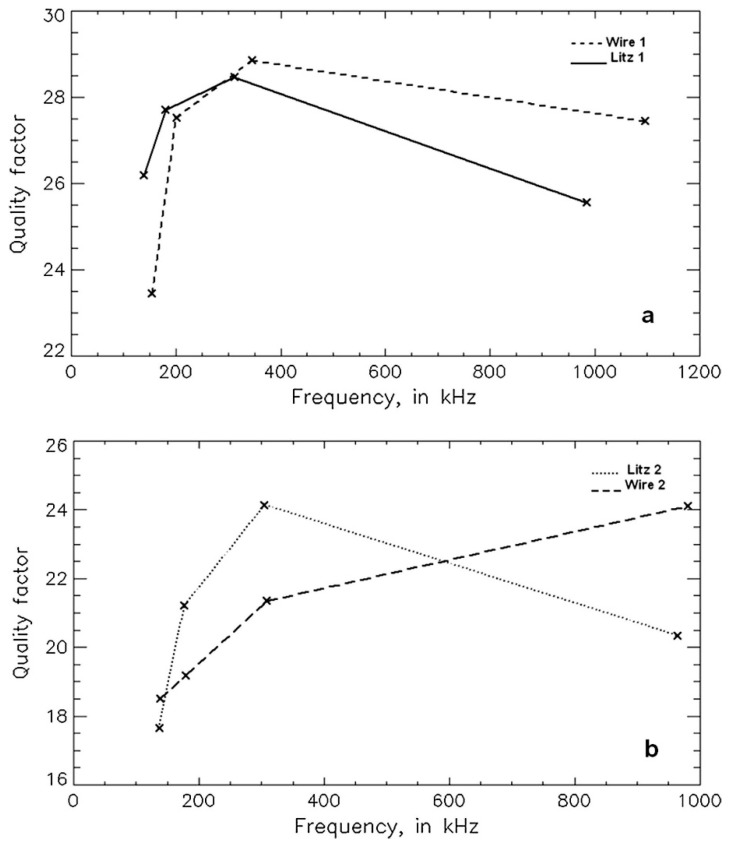
Quality factor measurement results as a function of the frequency: (**a**) 4 mm diameter wire (Wire 1) and 600 strands/0.10 mm diameter/3.45 mm total diameter Litz wire (Litz 1) and (**b**) 1.5 mm diameter wire (Wire 2) and 42 strands/0.18 mm diameter/1.75 mm total diameter Litz wire (Litz 2). Reprinted with permission from Ref. [53]. 2017, Elsevier.

According to the literature, Litz wires are particularly effective when the coil losses outweigh the sample losses. In this condition, with the use of high-quality capacitors, coil losses consist mainly of conductor losses. According to the literature, for frequencies of several hundred KHz (below 500 KHz), Litz wires help reduce losses compared to conventional wires (usually copper solid wires), whereas, for greater frequencies, the benefit provided by Litz wires is greatly reduced [52,53], as shown in Figure 9.

Therefore, MRI studies where the noise is expected to be coil-dominated could likely benefit from the use of Litz wires. Possible applications include low- and ultra-low-field MRI [51,52,54], low-field MRI with hyperpolarized contrast agents or pre-polarized MRI [42,43,44,45], as well as studies using coils of reduced dimensions, such as small animal MRI or parallel imaging using arrays of very small coils [44]. Litz wires have also been used for the construction of a coil dedicated to the measure of the hysteresis loops of magnetic samples in hyperthermia studies [55]; in addition, Litz wires can potentially be applied in the design of heating devices based on RF coils in magnetic fluid hyperthermia treatments [53].

### 9.2. Cooled Coils

One of the possible ways to minimize conductor losses is to cool the coil by exploiting the fact that the mean square thermal voltage fluctuations (Johnson noise) of the charge carriers in the conducting material can be reduced by lowering the coil temperature, which, consequently, allows a SNR increase. RF coil cooling has been proposed and employed by different researchers. For example, Wright and colleagues [56] demonstrated that cooling a small circular copper surface coil provided significant SNR gains for MR microimaging of a tissue structure in vivo. In particular, they designed and constructed a simple, cost-effective and easy-to-use liquid nitrogen vacuum Dewar to be used for microimaging at 1.5 T. The vacuum Dewar was constructed entirely using PVC plastic and was designed to maintain a 30 cm^3^ bath of liquid nitrogen (LN_2_). A high vacuum was obtained with an external pump disconnected from the Dewar before placement in the scanner. To reduce the loading effects, the surfaces were coated with an aluminized Mylar film. The vacuum obtained was sufficient to provide thermal insulation, so the external surface coil became slightly cooler. To illustrate the performance of the coil using in vivo imaging, a GE Medical Systems Signa 1.5-T whole body scanner and a receive coil with an outer diameter of a 17 mm loop with a chip capacitor and decoupled from the body coil transmission by a parallel chip crossed diode were used. MR microimaging in humans and rabbits demonstrated that, by cooling a small surface coil with liquid nitrogen, the SNR gain reached 2.7.

Cryogenically cooled coils produced by Bruker and Varian and realized with cryoprobes for high-resolution NMR of liquid samples with 3–5 mm diameters provided SNR gains with a factor of four [57].

The design and construction of a receive-only liquid nitrogen (LN_2_)-cooled coil and a cryostat system suitable for medical imaging on a 3T scanner was presented by Hu et al. [58]. The copper coil design was optimized by means of a computational electromagnetic (EM) simulation varying the coil sizes to achieve the best SNR.

The design of a nonmagnetic cryostat for LN_2_ comprised a homemade rectangular cooling unit that was released in polytetrafluoroethylene. To permit the circulation of LN_2_, three tubes were positioned to form a U-shaped path. The presence of the tube (tube 1 connected to the base of the LN_2_ reservoir and tube 2 for the circulation of LN_2_) made a difference in the pressure that permitted the vacuum pressure inside the cryostat to be below 0.8 mbar, where a 90 K final cooling temperature was achieved.

The performances of the system were evaluated by measuring and comparing the SNR of the acquired MR images by using a 3T scanner for three different applications (murine brain, hind legs and liver images) of the coil at room temperature and at cryogenic temperature. Furthermore, the SNR was calculated at different temperatures. The results showed an improvement 1.7-fold of the SNR gain with the cryogenic coil.

Sanchez et al. [59] presented a cryogenic coil setup for hyperpolarized ^13^C MRSI at 3T (32.13 MHz), which was an interesting case due to the nonrecoverable magnetization of the hyperpolarized injected compound. They developed a LN_2_-based cryogenically cooled coil setup that avoided the use of a cryocooler and any additional hardware inside the scanner during the experiments. Therefore, the cryogenic setup cooled in less than 1 h and was then used for a whole scan session (of over 12 h) in a small animal scanner. The system used a vacuum-insulated fiberglass cryostat that cooled a cold finger where the coil was attached. The coil was a multipurpose octagonal surface design for rodents. The coil and the preamplifier were cooled to 88 K and 77 K, respectively. The authors measured the Q factors ratio as Q_88K_/Q_290K_ = 550/280 and demonstrated that, in phantoms, a two-fold SNR enhancement was achieved. They also applied the technique during in vivo experiments and demonstrated the suitability of a cryogenic coil setup in ^13^C hyperpolarized metabolic experiments in healthy rats. The system proposed by the group seems to be beneficial for ^13^C metabolic imaging, but this needs to be proven with other experiments.

### 9.3. Superconducting Coils

The cryogenic RF coils proposed in the previous section demonstrated increasing the SNR performance in MRI experiments with small animals compared with conventional RF coils at room temperature, but, until now, there have been limitations for the safety and comfort of patients when applying such cryogenic probes to in vivo medical imaging.

Several papers have also proven that, with a superconducting probe, a great improvement in SNR is possible. Therefore, this technology was proposed by Bednorz and Muller in 1986 [60] and seems to have great potential in providing better SNR improvements for MRI applications. Shortly after, a cryogenically cooled high-temperature superconductor (HTS) surface coil was used for in vivo mouse brain acquisition in horizontal bore magnets, which showed a gain by a factor of 2.4 in SNR compared with a 20 mm quadrature room temperature surface coil at 9.4 T [61].

**Figure 10:**
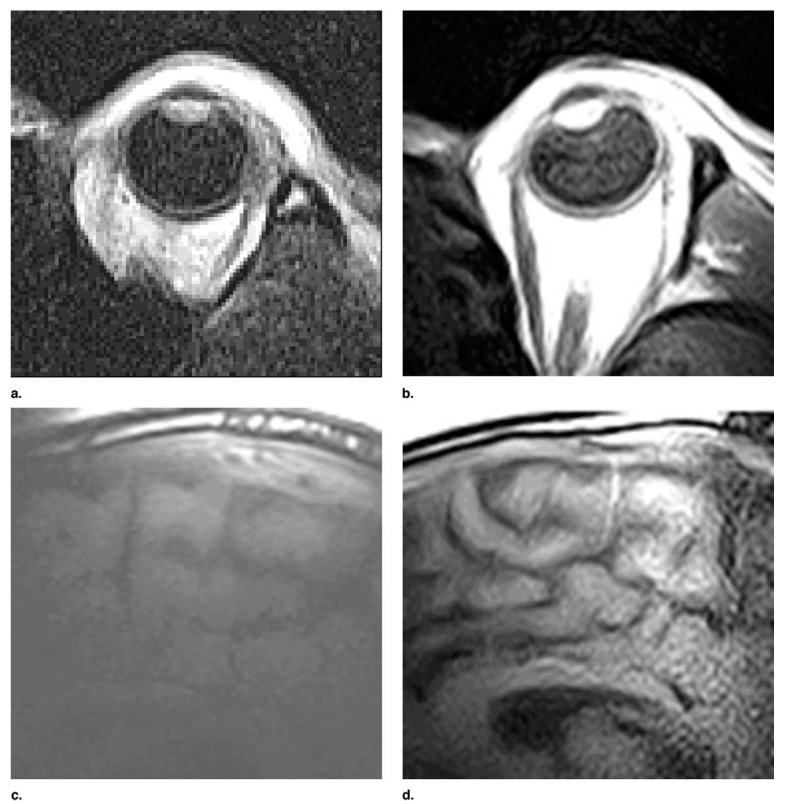
Human imaging comparisons. Images acquired at 0.2 T with same-sized room temperature copper coils: orbit (**a**) and brain (**c**) images acquired at 0.2 T with a 7.62 cm HTS coil and an orbit (**b**) and brain (**d**). Reprinted with permission from Ref. [62]. 2003, Elsevier.

Ma et al. [62] designed and built HTS surface coils constructed from 7.62 cm YBa_2_Cur_3_O_7_- thin films on a LaAlO_3_ substrate and cooled in a liquid nitrogen cryostat for a low-field MRI scanner. The coil tested included an interdigitated design and a spiral design. The interdigitated coil design consisted of a single inductive circuit with two turns, while the spiral coil design used a multi-turn simple spiral with a constant separation between turns. They also designed and built a cryostat system to house the HTS coils. Liquid nitrogen was used to maintain the temperature of the coil assembly at 77 °K. To quantify the performance of the HTS coil, a copper surface coil was made with the same outer diameter as the HTS coil from high Q capacitors and multi-turn copper wire. They measured the SNR gain results of 2.8-fold and 1.4-fold in images of a phantom acquired with an HTS coil versus a room temperature copper coil and a liquid nitrogen-cooled copper coil, respectively. They also acquired imaging of the subjects’ brains showing an increase in SNR and a reduction of the scan time at a low field, demonstrating that the development of this technology has promising benefits in diagnostic and therapeutic applications. Figure 10 shows a comparison between images acquired with copper and HTS coils.

Gogola et al. [63] designed and built a volume HTS receiving coil for a low-field MRI system. The coil was built as a single loop of thin tape of bismuth strontium calcium copper oxide with a width of 2.6 mm. The HTS coil, cooled to 77 K, was compared with uncooled and cooled copper coils, demonstrating an improvement in the SNR (SNR = 14.53 at room temperature, SNR = 38.75 for the cooled copper coil and SNR = 52.9 for the HTS coil), with the conclusion that the HTS materials seemed to be suitable for the construction of different kinds of coils for low-field applications and could be promising for high-field scanners as well.

Saniour et al. [64] presented a cryogenic free cryostat design to characterize the RF properties of a HTS. The vacuum chamber was a sealed in a four-way cross with a cold head installed at the bottom of the cross, and the coil being tested was placed inside the vacuum chamber at the top of the cross. Temperature measurements from 60 K to 300 K were provided with a precision of approximately 3 mK at 70 K, and the RF electrical power transmitted to the HTS coil ranged from 1 μW to 10 W. The measurements of the resonance frequency as a function of the temperature showed that the HTS coil could be fine-tuned through temperature control and the transition time was achieved in less than 12 μs, which was compatible with the MRI requirements.

Labbe et al. [65] presented an overview of the recent advances in the design of RF HTS coils and the potential applications of HTS coils and explained the technological hurdles that slow down their development and prospects for further improvements. Parts of these hurdles can be overcome with the development of MRI-compatible cryostats that avoid the use of liquid nitrogen and provide more easy-to-use and more user-friendly cryogen systems. Another problem that the scientific community is trying to solve is the imaging artefact induced by the interaction of a HTS coil and the static magnetic field B_0_ that can be mitigated at a low temperature by field-cooling the HTS coil in B_0_ at its working position in the MRI scanner. Another possible source of artefacts comes from RF field coupling between the coil used during transmission and the HTS reception coil that can be solved by implementing a decoupling strategy exploiting the nonlinearities in the electric responses of the HTS materials.

## 10. Novelties

### 10.1. Thin or Alternate Conductors

Barta et al. [66] tested the performance of thin or alternate conductors in contrast with conventional thick copper ones. Thinner conductors permit the construction of comfortable form-fitting coils made of flexible printed circuit board (PCB), which provides better SNR compared to rigid setups thanks to a closer proximity to the imaging region. Alternate conductors include aluminum, characterized by a conductivity of 3.54 × 10^8^ S/m and already employed in multimodality systems (X-ray MRI, MR LINAC and PET MRI) when interactions with ionizing radiation have to be minimized. Different 15 cm side square loops tuned at 20.56 MHz (^1^H frequency at 0.48 T) were built by using thin aluminum foil with different thicknesses (from 9 to 127 µm). Moreover, for comparison purposes, different typologies of copper conductors (tape, foil and bars) with variable thicknesses (from 17 to 600 µm) were employed for further coil building. At this tuning frequency, the skin depths for aluminum and copper were 18.1 μm and 14.4 μm, respectively. The coil workbench tests underlined that the coil quality factor improved when the conductor thickness increased and aluminum coils showed a lower efficiency compared to copper coils with similar thicknesses, as predicted theoretically. However, such measurements indicated that even the thinnest aluminum conductor coil (9 μm thickness) will provide an SNR that is 75% that of the 127 μm copper foil coil, although with a three-times higher coil resistance. SNR measurements performed on images acquired with different coils showed that copper coils built with 17 and 600 µm differ by only 20%, although there was a great difference in the thickness, while the 127 µm copper coil SNR result was only 35% better compared to the 9 µm aluminum coil one. These results showed that, although the use of conductors with a thickness of multiple skin depths certainly builds the best-performing RF coils, when using conductors which thicknesses are along the order of 1 skin depth, the losses increase only slightly. Finally, for thick coils built with similar thickness conductors, the performance provided by using aluminum was very similar to the one provided by copper, showing again that a significant increase in the coil resistance did not cause a dramatic SNR loss.

### 10.2. Flexible and Adaptive Coils

A traditional coil design very often deals with minimizing losses to provide a better SNR and improved coil performance. As previously reviewed, the use of conductors with a thickness much higher than the conductor skin depth in the fabrication process of RF coils made up of conventional solid wires (such as copper) helps to prevent skin losses and avoid SNR losses. In contrast, recent developments in the field of coil design have explored the use of thin or alternate conductors with the aim of building lightweight or flexible coils [66].

Flexible or even stretchable RF coil elements promote a more patient centered approach in the field of coil design by enabling the construction of RF coils that can better adapt to a patient’s anatomy, as well as to a wide range of patient sizes and shapes. Their final goal is to implement hardware customization to considerably improve clinical outcomes. Due to the optimization of the filling factor arising from their close proximity to the patient, this kind of coil provides a significant increase of the SNR and of the diagnostic image quality, together with improved patient comfort (which can also result in an improved image quality due to reduced motion artifacts) [67].

As a partial drawback, adaptable and flexible coils require the development of specific tuning and matching strategies to cope with the change in the electromagnetic properties consequent to the adaptation to a specific patient/anatomy [68].

**Figure 11:**
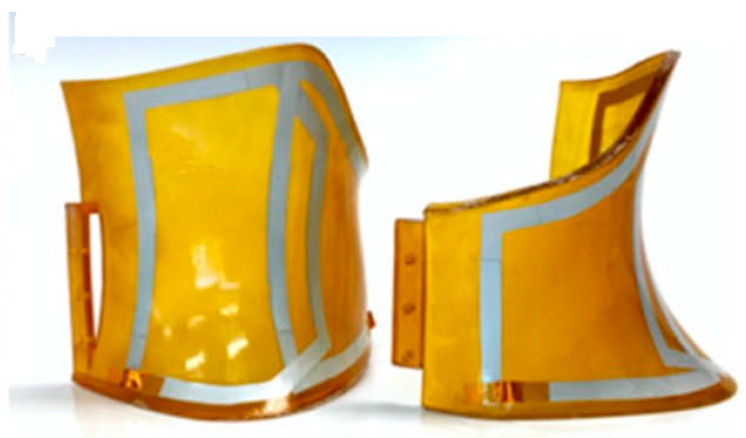
A custom coil array designed for covering the entire neck surface area. Reprinted from [67]. 2021, Springer.

Flexible coils have been tested in humans and are nowadays available in clinical settings, as reported, for instance, in the papers by Bae et al. [69], Wang et al. [70] and Zamarayeva et al. [67]. Figure 11 shows an example of a custom coil array.

Bae et al. [69] showed the performance of a highly flexible adaptive receive (AIR) anterior array coil for lung MRI using a zero echo time (ZTE) sequence at 3 T evaluated and compared with that of a conventional anterior array (CAA) coil. According to the results obtained from 66 patients, the AIR array coil provided a superior SNR, image quality and sharpness compared with the CAA coil; in addition, the combination of ZTE and AIR coils offered a better diagnostic capability, providing higher sensitivity and accuracy for lesion detection in cases of sub-centimeter nodules, emphysema and/or cysts than a ZTE-CAA coil. Further, the AIR array coil was more tolerated and even preferred over the CAA coil by 97% of the scanned patients.

In parallel, Wang and coworkers [70] proposed the design of a flexible coil based on an off-the-shelf conductor, suitable for MRI of the knee at 0.55 T (23.55 MHz), an environment where conductor losses can be significant over sample losses. The authors found that a highly effective “cable coil” could be obtained by resonating a commercially available RG-223 coaxial cable shield with a lumped capacitor while the inner conductor remained electrically floating. A 10 cm diameter cable coil provided enough flexibility to wrap around the knee and behaved similarly to a gold standard Cu-FR4 rigid copper loop in terms of the conductor loss and SNR. Moreover, the cable loops provide other benefits typical of coaxial cables, such as low cost, widespread availability, ease of assembly and high durability. The performance of a two-channel cable coil array was then evaluated in terms of tuning, decoupling and the SNR as a function of the geometry and overlap. According to the reported results, the two-channel cable coil array provided a good SNR robustness compared to the geometric variability, outperforming the two-channel coaxial coil array by 26 and 16% when the elements were overlapped by 20–40% or gapped by 30–50%, respectively. For in vivo testing on humans, a six-channel cable coil array was constructed that provided promising image quality, especially of the cartilage. In particular, incidental cartilage and bone pathologies were clearly defined in T1- and T2-weighted turbo spin echo images acquired in 3–4 min, suggesting that high-quality knee imaging is feasible in a clinically acceptable examination time.

The work by Zamarayeva and coworkers [67] described, for the first time, a novel approach for fabricating patient-specific MRI RF receive coil arrays using additive manufacturing. The process is based on the spray deposition of silver nanoparticle inks (which provide conductive traces) and dielectric materials (sprayed polystyrene) onto 3D-printed substrates to construct high-quality resonant circuits. Interestingly, the sample noise prevails over material losses in the proposed devices, suggesting their suitability for clinical settings. A printed four-channel neck array prototype for spine and carotid imaging was constructed in the paper, and its performance was compared to that of commercially available 2D four-channel neck arrays at 3 T. The printed array provided a higher SNR in phantom and superior-quality images in vivo of a volunteer compared to the conventional array coil, anticipating an improved diagnostic image quality. Indeed, the fabrication method proposed in this paper by producing high-quality imaging holds promise for the construction of customized or patient-specific 3D RF coils enabling an optimal fit to body parts with complex geometries.

Finally, novel opportunities for the development of adaptable RF coils arise from the use of wireless technology in signal transmission, which enables the elimination or reduction of cabling issues and simplifies coil handling. In this case, the picked-up signal is digitized before transmission near the RF coil; therefore, two of the main issues concern preserving the phase information of individual signals, as well as powering the active parts of coils [68].

### 10.3. Metamaterials

In high-field (>7 T) MRI examinations of the anatomical districts and tissue compartments, the dimensions are comparable to the wavelength associated with the magnetic field inside the tissue, resulting in significant distortions in the B_1_ transmit and receive fields and the formation of standing wave patterns. In this case, the use of new types of materials to be used as “inserts” to tailor the RF field pattern can provide significant improvements in the image quality over traditional RF coils. The development and applications of these novel materials were nicely reviewed in a recent paper by Webb et al. [71].

Among high-permittivity materials, the so-called “dielectric pads”, which can be placed around the body to shape RF distribution, provide quite a simple strategy to cope with B_1_^+^ inhomogeneity and to improve the quality of in vivo images. Proposed as an easier alternative to multi-transmit technology, dielectric pads are largely used in MRI. Materials with higher values of permittivity (*ε_r_* > 1000) that have proven to be useful for applications of clinical field strengths, specifically in brain imaging but also in cardiac studies, range from different metal titanate formulations (water solutions) to high-density ceramics such as zirconate titanate blocks. These types of materials not only increase the SNR by locally enhancing the transmit efficiency but also reduce the global maximum SAR, thus improving the safety. This peculiarity provides the opportunity to perform MRI studies in critical situations—for instance, in patients with a medical implant far from the region of interest for which safety concerns related to high-power deposition exist.

Being more flexible and lightweight, passive devices such as artificial dielectrics, metasurfaces, metamaterials and metamaterial-inspired structures represent a valid alternative to high-permittivity ceramic materials. Metamaterials originally defined a medium characterized by both negative permittivity and negative permeability in the same frequency range. More generally, the term “metamaterials” includes artificial composite materials, typically formed by subunits that are subwavelengths in dimension, that exhibit electromagnetic properties that cannot be found in naturally available materials. The solutions proposed in different MRI studies usually rely on the use of metasurfaces to produce highly effective permittivity materials following an integration approach; however, the possibility of producing very highly “effective permittivities” using purely conductive elements, so-called “artificial dielectrics”, has also been demonstrated.

As a further step, few studies have reported the use of these novel high-permittivity materials as resonating systems per se (so-called dielectric resonators), with the purpose of increasing B_1_ penetration while reducing the SAR. As reviewed in the paper by Webb et al. [71], different configurations have been tested at 7 T and 3 T, especially for imaging of the extremities, even if other anatomical districts (such as the breasts) have also been imaged.

Looking at the novelties in RF coil design, the so-called “traveling wave imaging” technology represents another promising approach in the context of high-field MRI when the body dimensions are of the same order of magnitude as the wavelengths inside tissues. Traveling wave imaging uses the MR scanner bore as a waveguide to support the propagation of an electromagnetic wave generated by a relatively small transceiver RF coil (e.g., a patch antenna), usually a cylindrical lining placed at one end of the MRI scanner. Accordingly, traveling wave imaging exploits far fields for signal excitation/reception, since the scanned subject is exposed to a traveling RF wave, emitted and received by the same antenna, for producing MR images [68,72].

In the context of unconventional materials for RF coil design, another interesting development concerns the use of carbon nanotubes (CNTs). CNT bundles provide significantly reduced skin effects at high frequencies compared to conventional metal conductors, which makes them attractive for the design of high-quality inductors. In particular, high electrical conductivity is expected in single-wall CNT networks due to minimum scattering of the charge transport. For instance, the results reported by Aly Saad Aly et al. [73] showed that CNTs do not suffer from skin effects, which occurs in similar Cu solid wires. In particular, surface coils constructed using CNTs showed a decrease in the resistance as the frequency increased, which approached that of conventional Cu coils at 300 MHz; moreover, a comparatively small inductance was found. In parallel, no significant differences in the inductance, impedance or quality factor were found among the CNT-coated and Cu solenoidal and small loop coils. The main disadvantage reported in this work concerning the use of CNTs in coil design is the high DC resistance, which can be overcome by investigating different CNT compositions, deposition methods, coil geometries and structures.

## 11. Conclusions

Coil losses are the dominant power loss mechanisms for low-frequency tuned coils; therefore, the design of an optimal coil is of particular importance for obtaining high-quality MR data.

At AC frequencies usually employed in MR, the coil conductor resistance is increased due to the skin effect, which distributes the current primarily on the conductor surface instead of uniformly over its cross-section, such as for the DC frequency. The conductor geometry strongly affects the coil performance and, consequently, the data SNR. In particular, the use of wire conductors obtains a better coil performance with respect to the strip conductor due to its more uniform current distribution, although it is difficult to handle such conductor typology, requiring qualified mechanical personnel for wire coil building.

After a brief description of the RF coil performance in terms of the quality indices and losses (and how these affect the data SNR), this review summarized the methods to estimate conductor losses in RF coils for MRI/MRS applications. In particular, the different theoretical and experimental approaches for estimating the skin effect losses in strip and wire conductors were reviewed, comprising analytical calculations and full-wave (FEM and FDTD) simulations. Moreover, a collection of papers regarding strategies for minimizing conductor losses (the use of Litz wire and cooled/superconducting coils, plus the novel emerging technologies) were briefly reviewed.

We believe that the materials reported in this review can provide a useful reference for RF coil designers, particularly for those interested in estimating or minimizing coil conductor losses in the MHz range.

## Figures and Tables

**Table 1:** Birdcage workbench test results.

Birdcage Conductor	*Q*	*r*	*η* (*µT*/*w*^1/2^)
Strip (*w* = 1 cm, *t* = 35 µm)	228	2.05	34.61
Strip (*w* = 1 cm, *t* = 800 µm)	374	2.33	42.74
Wire (*a* = 2.25 mm)	477	2.93	52.31

## Data Availability

Not applicable.
